# Differences in the treatment needs of patients with dementia with Lewy bodies and their caregivers and differences in their physicians’ awareness of those treatment needs according to the clinical department visited by the patients: a subanalysis of an observational survey study

**DOI:** 10.1186/s13195-024-01419-6

**Published:** 2024-03-14

**Authors:** Manabu Ikeda, Shunji Toya, Yuta Manabe, Hajime Yamakage, Mamoru Hashimoto

**Affiliations:** 1 Department of Psychiatry, Osaka University Graduate School of Medicine, 2-2 Yamadaoka, Suita-Shi, Osaka, 565-0871 Japan; 2 Medical Science, Sumitomo Pharma Co., Ltd., Chuo-Ku, Tokyo, Japan; 3 Department of Advanced Clinical Medicine, Division of Dementia and Geriatric Medicine, Kanagawa Dental University School of Dentistry, Yokosuka, Japan; 4 Insight Clinical Development Group, 3H Medi Solution Inc., Toshima-Ku, Tokyo, Japan; 5 Department of Neuropsychiatry, Kindai University Faculty of Medicine, Osakasayama, Osaka Japan

**Keywords:** Attending physicians, Caregivers, Clinical department, Dementia with Lewy bodies, Observational study, Patients, Subanalysis, Survey, Treatment needs

## Abstract

**Background:**

We investigated whether the treatment needs of patients with dementia with Lewy bodies (DLB) and their caregivers, along with their attending physicians’ perception of those treatment needs, differ according to the clinical department visited by the patients.

**Methods:**

This was a subanalysis of a multicenter, cross-sectional, observational survey study. Data from the main study were classified according to the clinical department visited by the patient: psychiatric group (P-group), geriatric internal medicine group (G-group), and neurology group (N-group). The treatment needs of patients and caregivers were defined as “the symptom that causes them the most distress”, and the frequency of each answer was tabulated.

**Results:**

This subanalysis included 134, 65, and 49 patient–caregiver pairs in the P-, G-, and N-groups, respectively. Statistically significant differences in patient background characteristics such as patient age; initial symptom domains; use of cholinesterase inhibitors, levodopa, antipsychotics, and Yokukansan; and total scores of the Mini-Mental State Examination, Neuropsychiatric Inventory-12, and Movement Disorder Society-Unified Parkinson’s Disease Rating Scale Parts II and III were shown among the three subgroups. While there were no differences in patients’ treatment needs among the subgroups, residual analysis showed that in the N-group, parkinsonism was more of a problem than other symptom domains (*p* = 0.001). There were significant differences in caregivers’ treatment needs among the three subgroups (*p* < 0.001). The patient–physician concordance rates for the symptom domains that caused patients the most distress were: P-group, 42.9% (kappa coefficient [κ] = 0.264); G-group, 33.3% (κ = 0.135), and N-group, 67.6% (κ = 0.484). The caregiver–physician concordance rates for the symptom domains that caused the caregivers the most distress were: P-group, 54.8% (κ = 0.351), G-group, 50.0% (κ = 0.244), and N-group, 47.4% (κ = 0.170).

**Conclusion:**

This subanalysis revealed differences in the treatment needs of patients with DLB and their caregivers according to the clinical department they attended. There might be a lack of awareness of those treatment needs by the attending physicians, regardless of their specialty.

**Trial registration:**

UMIN Clinical Trials Registry UMIN000041844.

**Supplementary Information:**

The online version contains supplementary material available at 10.1186/s13195-024-01419-6.

## Background

The number of patients with Alzheimer’s disease and other dementias is increasing with the growing aging population in Japan and other countries [1–3]. Dementia with Lewy bodies (DLB) is a common type of neurodegenerative dementia with clinical features that include cognitive fluctuations, recurrent visual hallucinations, parkinsonism, rapid eye movement sleep behavior disorder, and in some cases, delusions, autonomic dysfunction, sleep disturbances, and depression [4]. Therefore, patients with DLB are examined in various departments including psychiatry, neurology, geriatrics, and neurosurgery. Considering that the number of patients with dementia is increasing and the number of patients with DLB is also on the rise, it is expected that there will be more need to treat patients with DLB in various medical departments.

Depending on the initial symptoms experienced by a patient with DLB, the type of clinical department visited by the patients or caregivers may vary and is determined by the specialty of the attending physician. Of note, a patient with DLB can have multiple concurrent symptoms [5], and treatment strategies may differ depending on the specialty of the attending physician. A web survey of physicians attending patients with DLB that investigated treatment strategies among different departments demonstrated that psychiatrists were less likely to make parkinsonism a treatment priority, were less likely to prescribe levodopa as a first-line drug for parkinsonism, and were more likely to prescribe antipsychotics for psychiatric symptoms than neurologists and neurosurgeons [6, 7].

We previously conducted a questionnaire survey study in Japan to investigate the treatment needs of patients with DLB and their caregivers, and their physicians’ awareness of those treatment needs [8]. In this subanalysis, we aimed to investigate whether the background characteristics and treatment needs of patients with DLB and their caregivers differ according to the clinical department visited by the patients. We also investigated their attending physicians’ level of perception of those treatment needs in each clinical department.

## Methods

### Ethics approval and consent to participate

This study was approved by the Observational Study Ethics Committee of Osaka University Hospital (IRB No., 20171–4, November 11, 2020) and the ethics committee of each participating institution. This study was conducted in compliance with the ethical principles of the Declaration of Helsinki (revised in 2013), the Ethical Guidelines for Medical and Health Research Involving Human Subjects (partially revised in 2017), and the research protocol. This study was registered in the UMIN Clinical Trials Registry under the identifier UMIN000041844. Written informed consent was obtained from all patients and their caregivers. Physicians consented to participate in this study via the Internet.

### Study design and participants

This was a subanalysis of a multicenter, cross-sectional, observational survey study [8]. The study participants included patients with DLB and their caregivers (hereinafter referred to as patient–caregiver pairs) and their physicians. The study was conducted in Japan from September 2020 to July 2021 at 35 sites with physicians who are experts in DLB.

The main selection criteria for patients with DLB were patients with probable DLB based on the 2017 consensus report of the DLB Consortium [4], who were aged 50 years or older, whose attending physician had been in practice for more than 3 months, and who were outpatients and had a caregiver. Exclusion criteria were patients with Parkinson’s disease with dementia (if parkinsonism had been present for more than 1 year prior to the onset of dementia), patients who were not followed-up by an attending physician for more than 3 months prior to obtaining consent, and patients deemed inappropriate for inclusion by the attending physician due to inability to complete the questionnaire irrespective of their caregiver’s assistance.

The caregivers had to be at least 20 years of age and they had to be the primary caregiver of the DLB patient. The attending physician was defined as a physician who is an expert in DLB treatment in Japan; a detailed definition of who was considered an expert physician has been published previously [8].

### Assessment

After consent was obtained, patients and caregivers underwent the following assessments: the Japanese version of the MMSE (MMSE-J) [9] for cognitive impairment, the Japanese version of the Movement Disorder Society-Unified Parkinson’s Disease Rating Scale (MDS-UPDRS) [10] Parts II and III for activities of daily living and parkinsonism, the Japanese version of the Neuropsychiatric Inventory-12 (NPI-12) [11] for behavioral and psychological symptoms of dementia, the Cognitive Fluctuation Inventory [12] for cognitive fluctuation, and a shortened Japanese version of the Zarit Caregiver Burden Interview [13] for caregiver burden.

### Contents of the questionnaire

In the main study, a questionnaire survey was conducted to investigate which symptoms caused the patient/caregiver the most distress. For this purpose, 52 symptoms that are frequent and clinically important in DLB were pre-selected in the main study, and they were further classified into seven symptom domains as previously reported [8]. The contents of the questionnaire for the patients, caregivers, and attending physicians; the survey methodology using the questionnaire; and definition of each symptom have already been reported in detail in the primary study paper [8].

### Population to be analyzed in this subanalysis

For this subanalysis, we classified 263 patient–caregiver pairs in the full analysis set into the following three groups according to the clinical department visited by the patients, excluding pairs who visited a neurosurgery or general internal medicine department: psychiatric group (P-group), geriatric internal medicine group (G-group), and neurology group (N-group).

### Outcome

Patient characteristics, such as time since diagnosis of DLB, initial symptom domain, time since initial symptom domain onset, patients’ current symptoms, prescribed medications, and some assessment data were collected and compared among the three groups.

Symptom domains in patients with DLB were defined as follows: for cognitive impairment, patients recorded as having “cognitive impairment” (memory impairment, disorientation, executive dysfunction, attention dysfunction, fluctuating cognition, visuospatial dysfunction, other cognitive impairment) in the physician’s response to the question regarding the patient’s symptoms; for parkinsonism, those with a total score of > 7 or an individual score of > 2 in the following five MDS-UPDRS Part III subitems (resting tremor of the hands [3.15], kinetic tremor of the hands [3.16], facial expression [3.2], global spontaneity of movement [body bradykinesia] [3.14], and rigidity [3.3]); for psychiatric symptoms, those with a total score of ≥ 1 in the NPI-10, excluding the subitems of “night-time behavior” and “appetite” from the NPI-12; for eating behavior-related problems, those with a score of ≥ 1 for “appetite” in the NPI-12; for sleep-related disorders, those with a score of ≥ 1 for “night-time behavior” in the NPI-12; for autonomic dysfunction, those recorded as having “autonomic dysfunction” (orthostatic hypotension, disturbance of sweating, constipation, night-time dysuria, daytime dysuria, syncope, dizziness) in the physician’s response to the question regarding the patient’s symptoms; and for sensory disorders, those recorded as having “sensory disorder” (dysosmia) in the physician’s response to the question regarding the patient’s symptoms.

Patients’ and caregivers’ treatment needs were defined as the patients’ symptoms causing the patient or caregiver the most distress. Symptoms were tabulated and reclassified into seven symptom domains (cognitive impairment, parkinsonism, psychiatric symptoms, eating behavior-related problems, sleep-related disorder, autonomic dysfunction, and sensory disorder). To evaluate the differences in patients’ and caregivers’ treatment needs among the three groups, we compared the percentages in each domain.

Regarding the attending physicians’ perception of patients’ and caregivers’ treatment needs, we calculated the concordance rates between patients and their attending physicians and between caregivers and the attending physicians for the symptom domain causing the patient or caregiver the most distress. We also calculated concordance rates by each symptom domain that the patients or caregivers selected. The concordance rate was defined as the number of matched patient–physician or caregiver–physician pairs/number of patient–physician or caregiver–physician pairs in the analysis × 100 for each outcome. In calculating the concordance rate, the total number of patient–caregiver pairs in the analysis was defined as the number of case pairs in which both parties (e.g., patient and physician) had valid responses (excluding multiple responses to unanswered questions or single responses) to the questionnaires.

### Statistical analysis

Descriptive statistics were used to evaluate the background characteristics of the study participants and the symptom and symptom domain causing the patients or caregivers the most distress. Summary statistics were calculated as mean ± standard deviation for continuous variables and frequency and percentage for nominal variables. Point estimates and 95% confidence intervals (CIs) were calculated for each concordance rate, and kappa coefficients (κ) were calculated as a measure of agreement. Kappa coefficients were determined as follows: less than 0.4 indicated poor agreement; 0.4 to 0.6, moderate; 0.6 to 0.8, good; and 0.8 or more, excellent. In three-group comparisons, one-way analysis of variance was used for continuous data, and Fisher’s exact test was used for frequencies and percentages. Comparative and residual analyses for symptom domains that patients and caregivers selected were conducted using the chi-square test. The chi-square test for residual analysis was conducted with Bonferroni correction. The statistical significance level in this study was set at 0.05 (two-sided), and all analyses were conducted using Statistical Analysis Software ver. 9.4 (SAS Institute Inc., Cary, NC, USA).

## Results

**Fig. 1:**
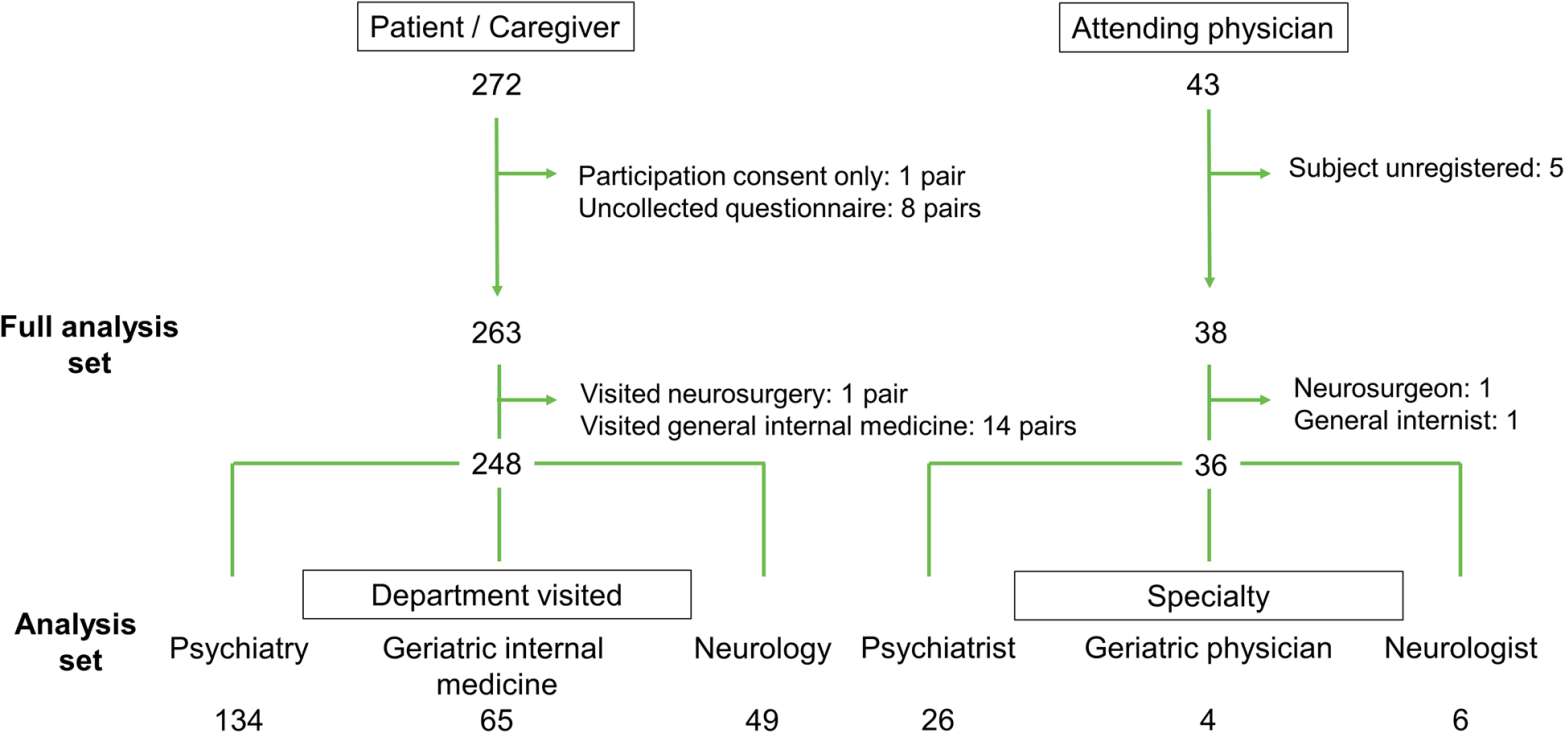
Disposition of the study participants

In total, 272 patient–caregiver pairs and 43 physicians provided consent to participate in the main study. Among the 263 patient–caregiver pairs in the full analysis set, one pair who visited a neurosurgery department and 14 pairs who visited a general internal medicine department were excluded from the subanalysis set. A total of 248 patient–caregiver pairs (P-group: 134, G-group: 65, N-group: 49) and 36 physicians (P-group: 26, G-group: four, N-group: six) were included in the subanalysis set (Fig. 1).

### Participant characteristics

The background characteristics of patients with DLB according to the clinical department subgroups are shown in Table 1. Patient age was significantly higher in the P-group and G-group vs the N-group (*p* < 0.001). The most frequent initial symptom domains were cognitive impairment in the P-group (38.8%) and G-group (58.5%), and parkinsonism was highest in the N-group (61.2%), with significant differences among the three groups for all symptom domains (*p* < 0.001). At the time of the study, symptoms of cognitive impairment were less frequent in the N-group vs the other two clinical department groups (P-group, 99.3%; G-group, 100%; N-group, 49.0%; *p* < 0.001). However, there were no significant differences in the time since initial symptom domain onset and the time since diagnosis of DLB among the three groups.

**Table 1:** Background characteristics of patients with DLB according to clinical department subgroups

	**P-group** **(*n* = 134)**	**G-group** **(*n* = 65)**	**N-group** **(*n* = 49)**	**Total** **(*N* = 248)**	***p*-value**
Patient’s age (y)	79.5 ± 6.7	80.9 ± 5.8	76.0 ± 6.7	79.2 ± 6.7	< 0.001
Patient’s sex (M/F)	66 / 68	25 / 40	31 / 18	122 / 126	0.032
Patient’s education history (y)	11.7 ± 3.0	11.0 ± 2.2	12.8 ± 2.9	11.8 ± 2.9	0.005
Institute					
University hospital	66 (49.3)	20 (30.8)	37 (75.5)	123 (49.6)	< 0.001
Non-university hospital	38 (28.4)	1 (1.5)	12 (24.5)	51 (20.6)	
Clinic	30 (22.4)	44 (67.7)	0 (0.0)	74 (29.8)	
Patient’s initial symptom domain					
Cognitive impairment	52 (38.8)	38 (58.5)	6 (12.2)	96 (38.7)	< 0.001
Parkinsonism	8 (6.0)	0 (0.0)	30 (61.2)	38 (15.3)	
Psychiatric symptoms	45 (33.6)	10 (15.4)	10 (20.4)	65 (26.2)	
Eating behavior-related problems	1 (0.7)	0 (0.0)	0 (0.0)	1 (0.4)	
Sleep-related disorder	18 (13.4)	12 (18.5)	2 (4.1)	32 (12.9)	
Autonomic dysfunction	2 (1.5)	4 (6.2)	1 (2.0)	7 (2.8)	
Sensory disorder	6 (4.5)	1 (1.5)	0 (0.0)	7 (2.8)	
Unknown	2 (1.5)	0 (0.0)	0 (0.0)	2 (0.8)	
Time since initial symptom domain onset					
Less than half a year	2 (1.5)	1 (1.5)	4 (8.2)	7 (2.8)	0.059
More than half a year but less than 1 year	9 (6.7)	14 (21.5)	3 (6.1)	26 (10.5)	
More than 1 year but less than 3 years	29 (21.6)	16 (24.6)	12 (24.5)	57 (23.0)	
More than 3 years but less than 5 years	38 (28.4)	15 (23.1)	10 (20.4)	63 (25.4)	
More than 5 years	49 (36.6)	18 (27.7)	18 (36.7)	85 (34.3)	
Unknown	7(5.2)	1 (1.5)	2 (4.1)	10 (4.0)	
Time since diagnosis of DLB (m)	28.7 ± 24.6	27.7 ± 27.3	39.3 ± 42.4	30.5 ± 29.7	0.072
Patient’s symptom domain					
Cognitive impairment	133 (99.3)	65 (100.0)	24 (49.0)	222 (89.5)	< 0.001
Parkinsonism	102 (76.1)	46 (70.8)	43 (87.8)	191 (77.0)	0.096
Psychiatric symptoms	116 (86.6)	57 (87.7)	38 (77.6)	211 (85.1)	0.250
Eating behavior-related problems	35 (26.1)	24 (36.9)	7 (14.3)	66 (26.6)	0.025
Sleep-related disorder	59 (44.0)	19 (29.2)	13 (26.5)	91 (36.7)	0.033
Autonomic dysfunction	64 (47.8)	30 (46.2)	12 (24.5)	106 (42.7)	0.015
Sensory disorder	16 (11.9)	4 (6.2)	7 (14.3)	27 (10.9)	0.327
Concomitant drugs					
Cholinesterase inhibitor	119 (88.8)	59 (90.8)	14 (28.6)	192 (77.4)	< 0.001
Memantine	19 (14.2)	17 (26.2)	9 (18.4)	45 (18.1)	0.121
Levodopa	47 (35.1)	10 (15.4)	40 (81.6)	97 (39.1)	< 0.001
Daily dose of levodopa	244.5 ± 149.5	310.0 ± 237.8	383.3 ± 237.3	286.0 ± 193.5	0.052
	(*n* = 41)	(*n* = 10)	(*n* = 15)	(*n* = 66)	
Zonisamide	9 (6.7)	3 (4.6)	4 (8.2)	16 (6.5)	0.735
Dopamine agonist	7 (5.2)	3 (4.6)	7 (14.3)	17 (6.9)	0.070
Anticholinergics	3 (2.2)	0 (0.0)	0 (0.0)	3 (1.2)	0.275
Antipsychotics	29 (21.6)	4 (6.2)	1 (2.0)	34 (13.7)	< 0.001
Yokukansan	20 (14.9)	22 (33.8)	2 (4.1)	44 (17.7)	< 0.001
Other CNS drugs	37 (27.6)	11 (16.9)	8 (16.3)	56 (22.6)	0.121
Anti-constipation drugs	24 (17.9)	10 (15.4)	6 (12.2)	40 (16.1)	0.642
Anti-dysuria drugs	7 (5.2)	4 (6.2)	4 (8.2)	15 (6.0)	0.761
MMSE-J total score	19.9 ± 6.0	20.3 ± 5.5	24.0 ± 4.9	20.8 ± 5.9	< 0.001
NPI-12 total score	18.7 ± 17.5	15.3 ± 15.8	9.7 ± 13.4	16.0 ± 16.6	0.004
MDS-UPDRS Part III total score	23.1 ± 19.8	21.2 ± 19.3	31.9 ± 20.9	24.3 ± 20.2	0.012
MDS-UPDRS Part II total score	11.1 ± 10.3	7.6 ± 8.9	18.2 ± 11.4	11.6 ± 10.7	< 0.001
CFI score	2.5 ± 3.3	2.5 ± 2.6	1.6 ± 3.1	2.3 ± 3.1	0.151
J-ZBI_8 score	9.2 ± 6.5	7.1 ± 4.9	7.5 ± 7.2	8.3 ± 6.3	0.059
Caregiver’s age (y)	65.4 ± 12.4	63.2 ± 14.7	66.2 ± 11.4	65.0 ± 12.9	0.408
Caregiver’s sex (M/F)	32 / 102	23 / 42	16 / 33	71 / 177	0.190
Caregiver living with the patient, Yes	108 (80.6)	49 (75.4)	43 (87.8)	200 (80.6)	0.254
Caregiver’s time spent with the patient (h/d)	14.7 ± 8.8	13.0 ± 9.3	17.0 ± 8.0	14.7 ± 8.9	0.060

Data are mean ± standard deviation or *n* (%)

*p*-value: In three-group (P-, G-, and N- group) comparisons, one-way analysis of variance was used for continuous data, and Fisher’s exact test was used for frequencies and percentages

Other CNS drugs included antidepressants (amitriptyline, clomipramine, sertraline, escitalopram, fluvoxamine, paroxetine, duloxetine, mirtazapine, trazodone) and anxiolytics (lorazepam, clonazepam, clotiazepam, diazepam, etizolam, bromazepam, alprazolam, tandospirone)

Anti-constipation drugs included linaclotide, elobixibat, magnesium oxide formulations, polyethylene glycol formulations, sennosides, diphenyl, mosapride, Chinese herbal medicine excepting irritant laxatives, and other

Anti-dysuria drugs included imidafenacin, oxybutynin hydrochloride, fesoterodine fumarate, mirabegron, vibegron, and urapidil

*Abbreviations*: *CFI* Cognitive Fluctuation Inventory, *CNS* Central nervous system, *DLB* Dementia with Lewy bodies, *F* Female, *G-group* Geriatric internal medicine group, *J-ZBI_8* Japanese version of the Zarit Caregiver Burden Interview, *M* Male, *MDS-UPDRS* Japanese version of the Movement Disorder Society-Unified Parkinson’s Disease Rating Scale, *MMSE-J* Japanese version of the Mini-Mental State Examination, *N-group* Neurology group, *NPI-12* Japanese version of the Neuropsychiatric Inventory-12, *P-group* Psychiatry group

Significant differences were also observed in the frequency of eating behavior-related disorders, sleep-related disorders, and autonomic dysfunction. Cholinesterase inhibitors were prescribed less frequently in the N-group vs the other two groups (P-group, 88.8%; G-group, 90.8%; N-group, 28.6%; *p* < 0.001). Levodopa was prescribed less frequently in the P-group (35.1%) and G-group (15.4%) vs the N-group (81.6%) (*p* < 0.001). Antipsychotics were prescribed more frequently in the P-group, and Yokukansan (traditional Chinese medicine) was prescribed more in the G-group.

The results of assessments are also shown in Table 1. MMSE-J mean ± standard deviation total score, an index of cognitive function, was 19.9 ± 6.0 in the P-group, 20.3 ± 5.5 in the G-group, and 24.0 ± 4.9 in the N-group (*p* < 0.001). The mean ± standard deviation NPI-12 total score was 18.7 ± 17.5 in the P-group, 15.3 ± 15.8 in the G-group, and 9.7 ± 13.4 in the N-group (*p* = 0.004). The mean ± standard deviation MDS-UPDRS Part III total score, a measure of the severity of parkinsonism, was 23.1 ± 19.8 in the P-group, 21.2 ± 19.3 in the G-group, and 31.9 ± 20.9 in the N-group (*p* = 0.012). The mean ± standard deviation MDS-UPDRS Part II total score, a measure of activities of daily living, was proportional to the severity of parkinsonism and was significantly higher in the N-group vs the other two groups (*p* < 0.001). Supplementary Tables 1–4 (Additional file 1) show subitem data for these assessments. There were no significant differences in caregiver characteristics or care burden by clinical department (Table 1).

### Treatment needs of patients with DLB

The symptom domains and individual symptoms for each domain causing patients the most distress according to clinical department subgroups are summarized in Table 2 and Supplementary Table 5 (Additional file 1). Invalid answers (multiple answers, unanswered, or do not know) were given by 57 of the 134 (42.5%) patients in the P-group, 26 of the 65 (40.0%) patients in the G-group, and 12 of the 49 (24.5%) patients in the N-group (Table 2). The most frequently reported symptom domains causing patients the most distress in the P-group were cognitive impairment, parkinsonism, and autonomic dysfunction (22 [16.4%], 19 [14.2%], and 15 [11.2%] patients, respectively). In the G-group, the most frequently reported symptom domains were autonomic dysfunction, cognitive impairment, and parkinsonism (12 [18.5%], 11 [16.9%], and eight [12.3%] patients, respectively). In the N-group, the most frequently reported symptom domains were parkinsonism, autonomic dysfunction, and cognitive impairment (19 [38.8%], six [12.2%], and five [10.2%] patients, respectively). Of the 52 pre-selected symptoms, those reported to cause patients in the P-group the most distress were: memory impairment in 16 (11.9%) patients; constipation, nine (6.7%); bradykinesia/akinesia, eight (6.0%); visual hallucinations, six (4.5%); and day-night reversal, five (3.7%) (Supplementary Table 5, Additional file 1). In the G-group, symptoms reported to cause patients the most distress were: constipation in seven (10.8%) patients; memory impairment, five (7.7%); and night-time dysuria, three (4.6%). In the N-group, the symptoms reported to cause patients the most distress were: bradykinesia/akinesia in eight (16.3%) patients; other cognitive impairment, four (8.2%); and gait disturbance and constipation, three (6.1%) each.

**Table 2:** Symptom domains that caused patients the most distress according to clinical department subgroups

Symptoms domain	P-group (*n* = 134)	G-group (*n* = 65)	N-group (*n* = 49)	*p*-value (all groups)	*p*-value (P-group)	*p*-value (G-group)	*p*-value (N-group)
Cognitive impairment	22 (16.4)	11 (16.9)	5 (10.2)	0.116	0.282	0.573	0.067
Parkinsonism	19 (14.2)	8 (12.3)	19 (38.8)		0.143	0.132	0.001
Psychiatric symptoms	11 (8.2)	5 (7.7)	2 (4.1)		0.330	0.813	0.168
Eating behavior-related problems	2 (1.5)	1 (1.5)	2 (4.1)		0.639	0.775	0.401
Sleep-related disorder	8 (6.0)	2 (3.1)	3 (6.1)		0.398	0.382	0.922
Autonomic dysfunction	15 (11.2)	12 (18.5)	6 (12.2)		0.527	0.106	0.363
Sensory disorder	0 (0.0)	0 (0.0)	0 (0.0)		n.c	n.c	n.c
Invalid answer	57 (42.5)	26 (40.0)	12 (24.5)	-	-	-	-

*p*-value (all groups): chi-squared test; *p*-value (P-, G-, and N- group): residual analysis

*Abbreviations*: *G-group* Geriatric internal medicine group, *n.c.* not calculated, *N-group* Neurology group, *P-group* Psychiatry group

### Differences in patients’ treatment needs according to clinical department subgroups

There were no differences in patients’ treatment needs among the subgroups (Table 2). However, the results of the residual analysis showed that in the N-group, parkinsonism tended to be selected more often than other symptom domains (*p* = 0.001).

### Differences in the attending physicians’ perception of patients’ treatment needs according to clinical department subgroups

**Fig. 2:**
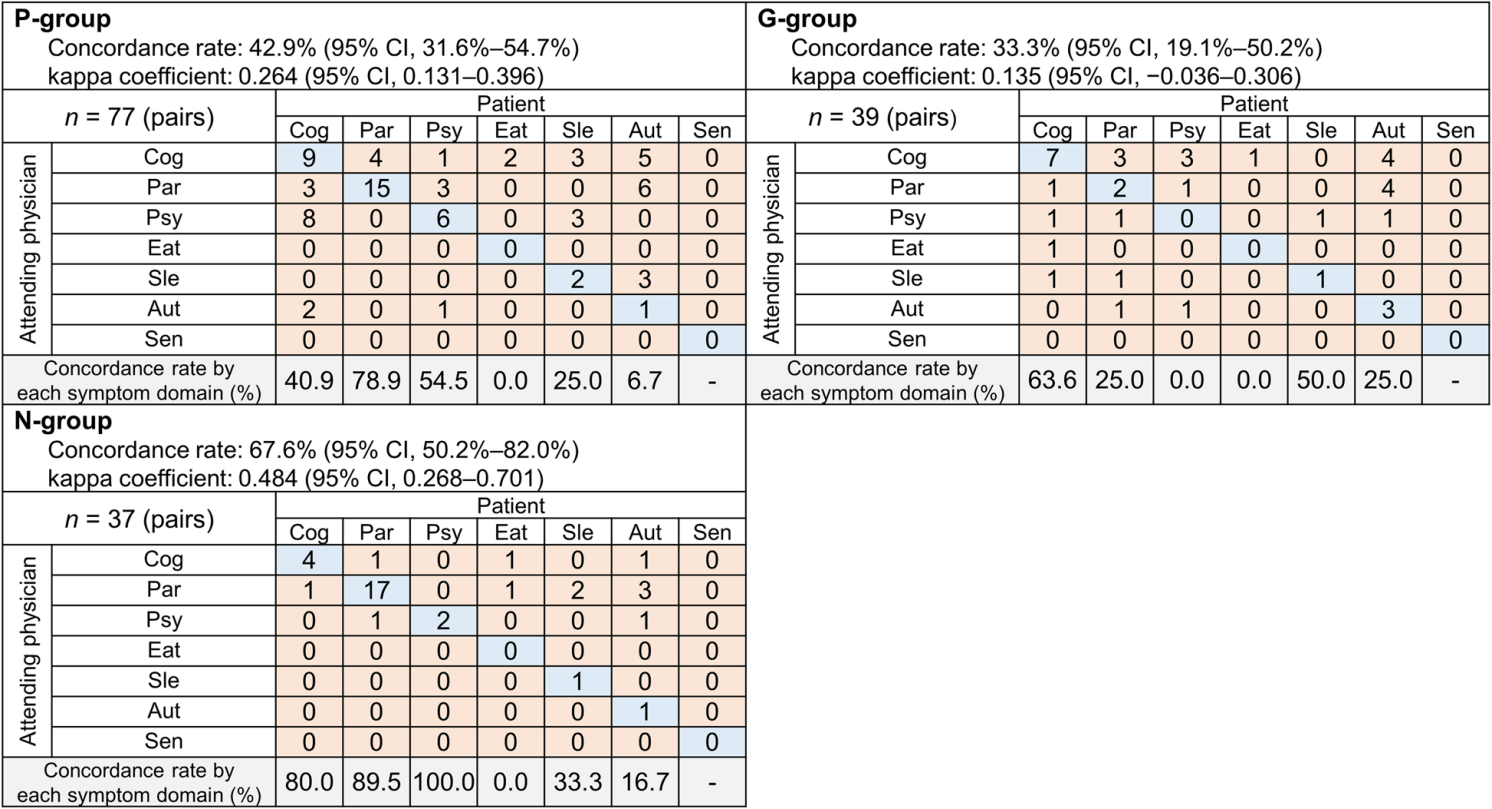
Symptom domains chosen by patients/physicians that caused patients the most distress by clinical department. Abbreviations: Aut, autonomic dysfunction; Cog, cognitive impairment; Eat, eating behavior-related problems; G-group, geriatric internal medicine group; N-group, neurology group; Par, parkinsonism; P-group, psychiatry group; Psy, psychiatric symptoms; Sen, sensory disorder; Sle, sleep-related problems

There were 77 patient–physician pairs in the P-group, 39 patient–physician pairs in the G-group, and 37 patient–physician pairs in the N-group, respectively. The patient–physician concordance rates for the symptom domains causing patients the most distress were 42.9% (95% CI 31.6%–54.7%, κ = 0.264 [95% CI: 0.131–0.396]) in the P-group, 33.3% (95% CI 19.1%–50.2%, κ = 0.135 [95% CI: –0.036–0.306]) in the G-group, and 67.6% (95% CI 50.2%–82.0%, κ = 0.484 [95%CI: 0.268–0.701]) in the N-group. Figure 2 shows the correlations between symptom domains chosen by patients and their attending physicians according to the clinical department subgroups. In the P-, G-, and N-groups, the concordance rates for cognitive impairment were 40.9%, 63.6%, and 80.0%; those of parkinsonism were 78.9%, 25.0%, and 89.5%; and those for psychiatric symptoms were 54.5%, 0.0%, and 100.0%, respectively. The concordance rates for eating behavior-related disorders, sleep-related disorder, and autonomic dysfunction were ≤ 50.0% in every group.

### Treatment needs according to caregivers

The symptom domains and individual symptoms for each domain that caused caregivers the most distress according to clinical department subgroups are summarized in Table 3 and Supplementary Table 6 (Additional file 1). Invalid answers were given by 50 of the 134 (37.3%) caregivers in the P-group, 17 of the 65 (26.2%) caregivers in the G-group, and 11 of the 49 (22.4%) caregivers in the N-group. Of the 52 pre-selected symptoms, in the P-group, those reported to cause caregivers the most distress were visual hallucinations in 12 (9.0%) caregivers; memory impairment, seven (5.2%); and bradykinesia/akinesia, six (4.5%) (Supplementary Table 6, Additional file 1). Among the seven symptom domains, those most frequently reported in the P-group as causing caregivers the most distress were psychiatric symptoms, cognitive impairment, and parkinsonism in 34 (25.4%), 23 (17.2%), and 14 (10.4%) caregivers, respectively (Table 3). In the G-group, the most frequently reported symptom domains were cognitive impairment, psychiatric symptoms, and autonomic dysfunction in 25 (38.5%), 12 (18.5%), and six (9.2%) caregivers, respectively. In the N-group, the most frequently reported symptom domains were parkinsonism, autonomic dysfunction, and psychiatric symptoms in 17 (34.7%), eight (16.3%), and six (12.2%) caregivers, respectively. In the G-group, symptoms reported to cause caregivers the most distress were memory impairment in 14 (21.5%), executive dysfunction in six (9.2%), and agitation/aggression in five (7.7%) caregivers. In the N-group, symptoms reported to cause caregivers the most distress were bradykinesia/akinesia in five (10.2%) and postural instability, constipation, and night-time dysuria in three (6.1%) caregivers each.

**Table 3:** Symptom domains that caused caregivers the most distress according to clinical department subgroups

Symptoms domain	P-group (*n* = 134)	G-group (*n* = 65)	N-group (*n* = 49)	*p*-value (all groups)	*p*-value (P-group)	*p*-value (G-group)	*p*-value (N-group)
Cognitive impairment	23 (17.2)	25 (38.5)	3 (6.1)	< 0.001	0.461	< 0.001	0.001
Parkinsonism	14 (10.4)	0 (0.0)	17 (34.7)		0.601	< 0.001	< 0.001
Psychiatric symptoms	34 (25.4)	12 (18.5)	6 (12.2)		0.006	0.321	0.025
Eating behavior-related problems	3 (2.2)	1 (1.5)	1 (2.0)		0.631	0.678	0.898
Sleep-related disorder	6 (4.5)	4 (6.2)	3 (6.1)		0.807	0.833	0.948
Autonomic dysfunction	4 (3.0)	6 (9.2)	8 (16.3)		0.015	0.611	0.017
Sensory disorder	0 (0.0)	0 (0.0)	0 (0.0)		n.c	n.c	n.c
Invalid answer	50 (37.3)	17 (26.2)	11 (22.4)	-	-	-	-

*p*-value (all groups): chi-squared test; *p*-value (P-, G-, and N- group): residual analysis

*Abbreviations*: *G-group* Geriatric internal medicine group, *n.c.* not calculated, *N-group* Neurology group, *P-group* Psychiatry group

### Differences in treatment needs according to caregivers by clinical department subgroups

The differences in caregivers’ treatment needs among the three subgroups were significant (*p* < 0.001). Residual analysis showed that psychiatric symptoms tended to be selected more frequently (*p* = 0.006) and autonomic dysfunction tended to be selected less frequently (*p* = 0.015) in the P-group than in the other two subgroups. Cognitive impairment also tended to be selected more frequently (*p* < 0.001) and parkinsonism tended to be selected less frequently (*p* < 0.001) in the G-group than in the other two subgroups. In the N-group, parkinsonism and autonomic dysfunction tended to be selected more frequently (*p* < 0.001 and* p* = 0.017) and cognitive impairment and psychiatric symptoms tended to be selected less frequently (*p* = 0.001 and *p* = 0.025) than in the other two subgroups.

### Differences in attending physicians’ perception of caregivers’ treatment needs according to clinical department subgroups

**Fig. 3:**
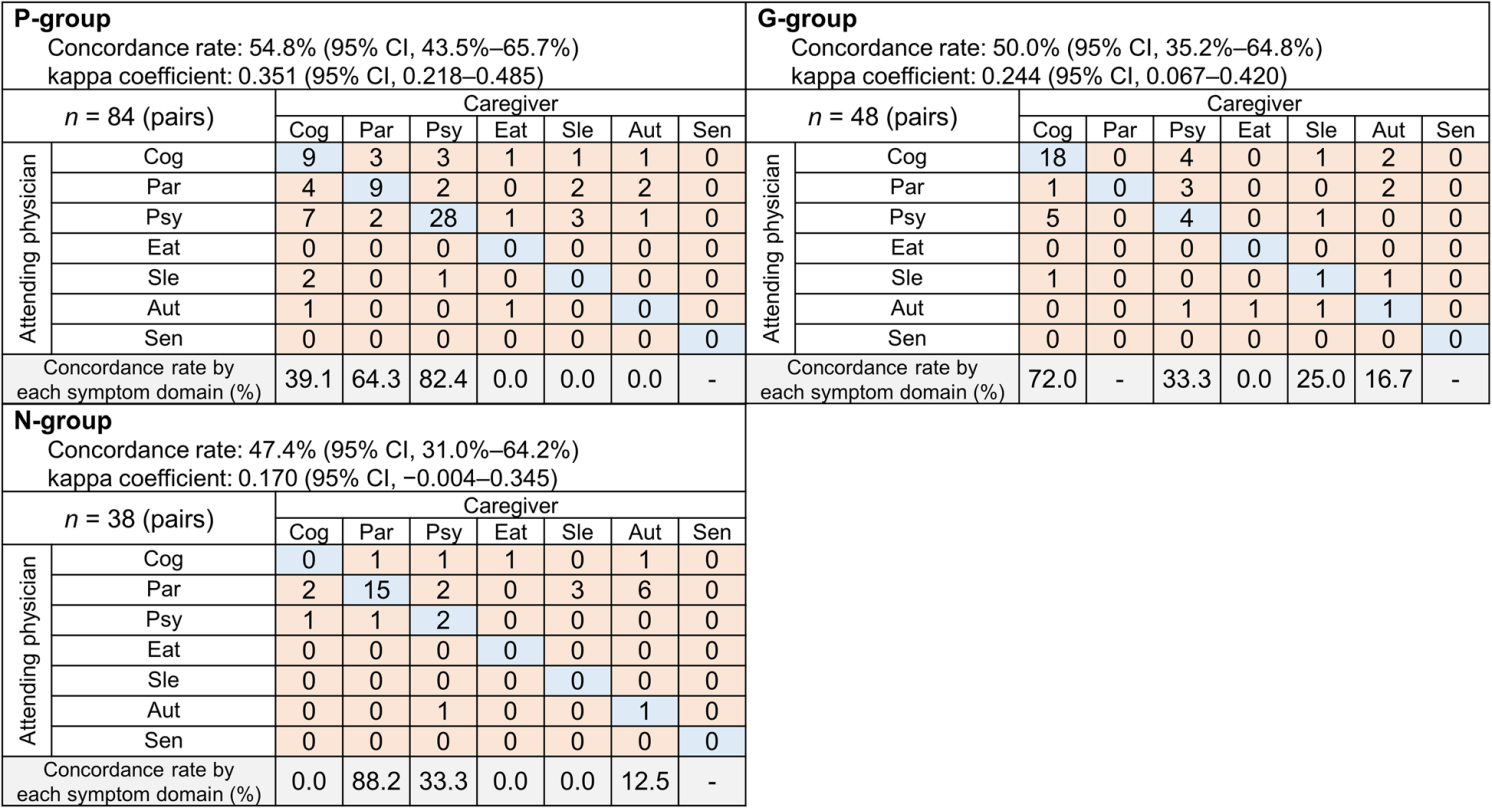
Symptom domains chosen by caregivers/physicians that caused caregivers the most distress by clinical department. Abbreviations: Aut, autonomic dysfunction; Cog, cognitive impairment; Eat, eating behavior-related problems; G-group, geriatric internal medicine group; N-group, neurology group; Par, parkinsonism; P-group, psychiatry group; Psy, psychiatric symptoms; Sen, sensory disorder; Sle, sleep-related problems

There were 84 caregiver–physician pairs in the P-group, 48 in the G-group, and 38 in the N-group. The caregiver–physician concordance rates for the symptom domains that caused the caregiver the most distress were 54.8% (95% CI 43.5%–65.7%, κ = 0.351 [95% CI: 0.218–0.485]) in the P-group, 50.0% (95% CI 35.2%–64.8%, κ = 0.244 [95% CI: 0.067–0.420) in the G-group, and 47.4% (95% CI 31.0%–64.2%, κ = 0.170 [95% CI: –0.004–0.345]) in the N-group. Figure 3 shows the correlations between symptom domains chosen by caregivers and the attending physicians according to clinical department subgroups. The concordance rates for cognitive impairment were 39.1%, 72.0%, and 0%; those for parkinsonism were 64.3%, not calculated, and 88.2%; and those for psychiatric symptoms were 82.4%, 33.3% and 33.3% in the P-group, G-group, and N-group, respectively. The concordance rate for eating behavior-related disorders, sleep-related disorder, and autonomic dysfunction were under 50% in every group.

## Discussion

To the best of our knowledge, this is the first report to examine the differences in background characteristics and treatment needs of patients with DLB and their caregivers, as well as the attending physicians’ awareness of their treatment needs, according to the clinical department visited by the patient.

In this study, there were no significant differences in the time since the initial symptom domain onset and the time since diagnosis of DLB among the three groups, while there were significant differences among the three subgroups in patients’ initial symptoms, severity of symptoms, and prescribed medications at the time of the survey. The majority of initial symptoms were cognitive dysfunction and psychiatric symptoms in the P-group; cognitive dysfunction, autonomic neuropathy, and psychiatric symptoms in the G-group; and parkinsonism and psychiatric symptoms in the N-group. The severity of cognitive dysfunction, psychiatric symptoms, and parkinsonism also differed among the three groups. The P- and G-groups had a higher severity of cognitive dysfunction and psychiatric symptoms than the N-group, and the N-group had a higher severity of parkinsonism than the other two groups. These results suggest that neurology departments may have more patients with DLB who are diagnosed with dementia within 1 year of presenting with parkinsonism, while psychiatry and geriatric departments may have more patients with DLB having cognitive dysfunction or psychiatric onset type. Thus, our results regarding the initial symptoms and their severity among the departments may be useful for future studies on clinical profiles such as disease progression and symptom severity in subtypes of DLB.

This subanalysis study revealed new findings regarding trends in prescribing medications by physician specialty. A previous study suggested differences in prescription rates of first-line medications for parkinsonism between general psychiatrists and neurologists, as well as differences in the prescription rates of antipsychotic medications for behavioral and psychological symptoms of dementia [6, 7]. In the present study, neurologists less frequently prescribed cholinesterase inhibitors for cognitive dysfunction than other specialists. Although cholinesterase inhibitors rarely worsen symptoms of parkinsonism, this worsening could theoretically be expected because of the alterations in the balance of acetylcholine and dopamine in the brains of patients with DLB [14]. For this reason, neurologists specializing in movement disorders may prescribe cholinesterase inhibitors less often than other specialists. However, DLB causes more damage to cholinergic nerves at muscarinic M1 receptors in the brain than Alzheimer’s disease [15], and donepezil, a cholinesterase inhibitor, is indicated in Japan for improvement of cognitive impairment in DLB [16, 17]. In the present study, neurologists prescribed levodopa for parkinsonism more frequently than psychiatry and geriatric specialists, which is consistent with a previous study that reported higher prescription rates of levodopa as a first-line therapy for parkinsonism in DLB among neurologists compared with psychiatrists [6]. Considering prescription rates of each parkinsonian in psychiatry and geriatric specialist, however, they might prescribe levodopa as first-line therapy for parkinsonism with DLB. The physicians in this study were experts in DLB, assuming that levodopa, rather than anticholinergics or dopamine agonists, has been becoming more common as the first-line drug. Furthermore, similar to a previous study [7], antipsychotics were prescribed less frequently in the N-group compared with the P-group, suggesting that neurologists may have fewer opportunities to prescribe antipsychotics for psychosis. Yokukansan was frequently prescribed in the G-group, and Yokukansan may be effective for neuropsychiatric symptoms according to a recent systematic review [18].

The treatment needs of patients and caregivers varied among the three clinical department subgroups. In fact, among the 52 pre-selected symptoms, patients with DLB in the P-group, G-group, and N-group selected 27 (51.9%), 21 (40.4%), and 21 (40.4%) symptoms, respectively, and caregivers in the P-group, G-group, and N-group selected 24 (46.2%), 17 (32.7%), and 22 (42.3%) symptoms, respectively. When the individual symptoms were recategorized into seven symptom domains, there were no statistically significant differences in the patients’ treatment needs among the three clinical department subgroups. However, it should be noted that the most frequently reported treatment need of patients in the N-group was parkinsonism, and the results of the residual analysis within the N-group showed that more patients selected parkinsonism than the other symptom domains (*p* = 0.001). Physicians reported that approximately 60% of the patients in the N-group had parkinsonism as the initial symptom domain. Therefore, it can be assumed that many patients with DLB who visit neurologists were troubled by symptoms of parkinsonism and visited neurologists to seek medical care because their symptoms had not been resolved. Parkinsonism is thought to be caused by dopamine depletion in the substantia nigra striatum, and dopamine replacement therapy is the main symptomatic treatment. A previous study that evaluated the progression of clinical features of patients with DLB showed that the UPDRS Part III score, a severity score of parkinsonism in patients with DLB, increased by 3.2 points in 6 months [19].

When categorized into seven symptom domains, caregivers’ treatment needs showed different trends among the three clinical department subgroups. The most frequently reported symptom domains by the caregivers were psychiatric symptoms (25.4%) in the P-group, cognitive impairment (38.5%) in the G-group, and parkinsonism (34.7%) in the N-group. The initial symptom domains commonly reported by the attending physician were also different among three groups; cognitive impairment (38.8%) and psychiatric symptoms (33.6%) in the P-group, cognitive impairment (58.5%) in the G-group, and parkinsonism (61.2%) in the N-group. Thus, the treatment needs of caregivers may be relatively dependent on the patient's initial symptom domain regardless of the clinical department subgroup. However, these results also indicated that while many of the treatment needs of patients and caregivers coincide, the treatment needs of a minority should not be ignored.

When evaluating the patient–physician and caregiver–physician concordance rates for the symptom domains causing patients and caregivers the most distress, all κ values for the three subgroups were less than 0.5 and 0.4, respectively. This indicates that the attending physicians’ perception of patients’ and caregivers’ treatment needs might be poor in all three clinical department subgroups, despite the fact that all attending physicians were experts in DLB. This also suggests that DLB symptoms are diverse, and regardless of the specialty of the attending physician or clinical department visited by the patient, DLB care is difficult, and the treatment needs of patients and caregivers cannot be easily met.

Based on the results of this study, to address the treatment needs of patients, attending physicians in the P-group should pay attention to cognitive dysfunction, and those in the G-group should pay attention to parkinsonism and psychiatric symptoms. In addition, to address the treatment needs of caregivers, it may be important for attending physicians in the P-group to pay attention to cognitive dysfunction, for attending physicians in the G-group to pay attention to psychiatric symptoms, and for attending physicians in the N-group to pay attention to cognitive dysfunction and psychiatric symptoms. Furthermore, the concordance rates for eating behavior-related problems, sleep-related disorders, and autonomic neuropathy were less than 50% in all departments, suggesting that attending physicians should pay more attention to these problems. This trend was consistent with the results of the overall study and was confirmed to be common regardless of the clinical department.

In summary, our results showed that psychiatry and geriatric specialists often treated patients with cognitive impairment and severe psychiatric symptoms, and that they often prescribed cholinesterase inhibitors, antipsychotics, and/or Yokukansan to treat these symptoms. These specialists sometimes prescribed levodopa for treating parkinsonism. Neurologists, however, often treated patients with more severe parkinsonism but relatively mild cognitive impairment and psychiatric symptoms, and they used levodopa and other antiparkinsonian medications for therapeutic intervention. The majority of the patients were treated in accordance with their initial symptom or treatment needs. However, the treatment needs of patients and caregivers are diverse, and careful attention to individual patients, especially with regard to sleep-related disorders and autonomic symptoms, may further contribute to the treatment needs of patients and caregivers.

### Limitations

The present study has some limitations, such as those inherent to questionnaire surveys. First, a literature search was conducted prior to commencing the study, but no articles discussing all the clinical manifestations of DLB could be found. Therefore, the 52 symptoms that were considered to be frequent and clinically important for patients with DLB were selected based on an internal discussion, so it is possible that some relatively important clinical symptoms may have been inadvertently omitted from this study. Second, patients who could respond to the survey were selected by their attending physicians. It is difficult to generalize the results of this study to all patients with DLB, especially because this study was limited to outpatients. Third, initial symptom domains and prescribed medications are assumed to have been checked or prescribed by the physicians, and we were not able to trace back to the actual medical record information. Fourth, the attending physicians who participated in this study were experts in DLB; thus, our results cannot be generalized to all physicians or medical facilities. Finally, patients with a sleep-related disorder were defined as those having an NPI-12 night-time behavior subitem score ≥ 1 in this study. Therefore, restless legs syndrome and periodic limb movement disorder were not included as a sleep-related disorder.

## Conclusions

In conclusion, this subanalysis study revealed differences in the treatment needs of patients and caregivers according to the practice attributes of their attending physicians, as well as a lack of awareness of those treatment needs by the attending physicians, regardless of their specialty. It is important to develop therapeutic agents and treatment strategies to meet the treatment needs of patients with DLB and their caregivers.

### Supplementary Information

**Additional file 1: Supplementary Table 1.** Scores for each item of the MMSE-J. **Supplementary Table 2.** Scores for each item of the NPI-12. **Supplementary Table 3.** Scores for each item of the MDS-UPDRS Part III. **Supplementary Table 4.** Scores for each item of the MDS-UPDRS Part II. **Supplementary Table 5.** Symptom domains and individual symptoms for each domain that caused patients the most distress. **Supplementary Table 6.** Symptom domains and individual symptoms for each domain that caused caregivers the most distress.

## Data Availability

Owing to participant privacy, individual-level data cannot be made publicly available.

## Abbreviations

CFI: Cognitive Fluctuation Inventory
CI: Confidence interval
DLB: Dementia with Lewy bodies
J-ZBI_8: Japanese version of the Zarit Caregiver Burden Interview
MMSE: Mini-Mental State Examination
MMSE-J: Japanese version of the MMSE
MDS-UPDRS: Movement Disorder Society-Unified Parkinson’s Disease Rating Scale
NPI-12: Neuropsychiatric Inventory-12
