# Supplementary material for: Differences in the treatment needs of patients with dementia with Lewy bodies and their caregivers and differences in their physicians’ awareness of those treatment needs according to the clinical department visited by the patients: a subanalysis of an observational survey study
Source: Alzheimers Res Ther. 2024 Mar 14;16:59. doi: 10.1186/s13195-024-01419-6 (PMC10938676; doi:10.1186/s13195-024-01419-6)
Supplement: Supplementary file 1 — Additional file 1: Supplementary Table 1. Scores for each item of the MMSE-J. Supplementary Table 2. Scores for each item of the NPI-12. Supplementary Table 3. Scores for each item of the MDS-UPDRS Part III. Supplementary Table 4. Scores for each item of the MDS-UPDRS Part II. Supplementary Table 5. Symptom domains and individual symptoms for each domain that caused patients the most distress. Supplementary Table 6. Symptom domains and individual symptoms for each domain that caused caregivers the most distress. [file 13195_2024_1419_MOESM1_ESM.docx]

**Additional file 1. Supplementary Materials**

**Supplementary Table 1.** Scores for each item of the MMSE-J

|  | **P-group**  **(*n* = 134)** | **G-group**  **(*n* = 65)** | **N-group**  **(*n* = 49)** | **Total**  **(*N* = 248)** | ***p*-value** |
| --- | --- | --- | --- | --- | --- |
| 1. Orientation to time | 3.1 ± 1.7 | 2.8 ± 1.9 | 4.1 ± 1.3 | 3.2 ± 1.8 | < 0.001 |
| 2. Orientation to place | 3.8 ± 1.4 | 3.7 ± 1.2 | 4.2 ± 1.0 | 3.8 ± 1.3 | 0.056 |
| 3. Registration | 2.6 ± 0.8 | 2.8 ± 0.6 | 2.8 ± 0.4 | 2.7 ± 0.7 | 0.093 |
| 4. Attention and calculation | 1.8 ± 1.6 | 2.2 ± 1.7 | 2.9 ± 1.8 | 2.1 ± 1.7 | 0.001 |
| 5. Recall | 1.2 ± 1.1 | 1.2 ± 1.2 | 1.8 ± 0.9 | 1.3 ± 1.1 | 0.002 |
| 6. Naming | 1.9 ± 0.3 | 1.9 ± 0.3 | 2.0 ± 0.0 | 1.9 ± 0.3 | 0.163 |
| 7. Repetition | 0.9 ± 0.3 | 0.9 ± 0.3 | 1.0 ± 0.1 | 0.9 ± 0.3 | 0.090 |
| 8. Comprehension | 2.4 ± 0.9 | 2.5 ± 0.9 | 2.7 ± 0.5 | 2.5 ± 0.8 | 0.118 |
| 9. Reading | 0.9 ± 0.3 | 0.9 ± 0.3 | 1.0 ± 0.1 | 0.9 ± 0.3 | 0.192 |
| 10. Writing | 0.7 ± 0.5 | 0.7 ± 0.4 | 0.8 ± 0.4 | 0.7 ± 0.4 | 0.103 |
| 11. Drawing | 0.6 ± 0.5 | 0.7 ± 0.5 | 0.7 ± 0.5 | 0.7 ± 0.5 | 0.363 |

Data are mean ± standard deviation.

*p*-value: one-way analysis of variance for three-group comparisons (P-, G-, and N- group).

Abbreviations: G-group, geriatric internal medicine group; MMSE-J, Japanese version of the Mini-Mental State Examination; N-group, neurology group; P-group, psychiatry group.

**Supplementary Table 2.** Scores for each item of the NPI-12

|  | **P-group**  **(*n* = 134)** | **G-group**  **(*n* = 65)** | **N-group**  **(*n* = 49)** | **Total**  **(*N* = 248)** | ***p*-value** |
| --- | --- | --- | --- | --- | --- |
| 1. Delusions | 1.7 ± 3.0 (125) | 1.4 ± 2.6 (65) | 0.6 ± 2.2 (49) | 1.4 ± 2.8 (239) | 0.080 |
| 2. Hallucinations | 2.5 ± 3.3 (128) | 1.3 ± 2.5 (65) | 1.6 ± 2.7 (49) | 2.0 ± 3.0 (242) | 0.020 |
| 3. Agitation | 1.5 ± 3.0 (129) | 1.1 ± 2.0 (65) | 0.8 ± 2.3 (49) | 1.3 ± 2.6 (243) | 0.295 |
| 4. Depression | 1.1 ± 1.9 (129) | 0.9 ± 1.6 (65) | 1.0 ± 1.8 (49) | 1.0 ± 1.8 (243) | 0.834 |
| 5. Anxiety | 1.8 ± 2.8 (130) | 1.4 ± 2.3 (65) | 0.7 ±1.8 (49) | 1.5 ± 2.5 (244) | 0.041 |
| 6. Euphoria | 0.3 ± 0.9 (130) | 0.3 ± 1.0 (65) | 0.0 ± 0.0 (49) | 0.3 ± 0.9 (244) | 0.070 |
| 7. Apathy | 3.5 ± 3.9 (129) | 3.0 ± 4.0 (65) | 1.4 ± 3.1 (49) | 2.9 ± 3.9 (243) | 0.007 |
| 8. Disinhibition | 0.3 ± 1.2 (126) | 0.6 ± 1.7 (65) | 0.3 ± 1.0 (49) | 0.4 ± 1.3 (240) | 0.447 |
| 9. Irritability | 1.7 ± 2.8 (127) | 1.0 ± 2.3 (65) | 1.2 ± 2.7 (49) | 1.4 ± 2.7 (241) | 0.265 |
| 10. Aberrant motor behavior | 1.0 ± 2.3 (129) | 1.0 ± 2.7 (65) | 0.2 ± 0.6 (49) | 0.8 ± 2.2 (243) | 0.054 |
| 11. Night-time behavior | 2.5 ± 3.6 (131) | 1.7 ± 2.9 (64) | 1.4 ± 3.0 (49) | 2.0 ± 3.3 (244) | 0.082 |
| 12. Appetite | 1.4 ± 2.7 (130) | 1.6 ± 2.8 (62) | 0.5 ± 1.5 (49) | 1.3 ± 2.6 (241) | 0.066 |

Data are mean ± standard deviation (*n*).

*p*-value: one-way analysis of variance for three-group comparisons (P-, G-, and N- group).

Abbreviations: G-group, geriatric internal medicine group; N-group, neurology group; NPI-12P, Japanese version of the Neuropsychiatric Inventory-12; P-group, psychiatry group.

**Supplementary Table 3.** Scores for each item of the MDS-UPDRS Part III

|  | **P-group**  **(*n* = 134)** | **G-group**  **(*n* = 65)** | **N-group**  **(*n* = 49)** | **Total**  **(*N* = 248)** | ***p*-value** |
| --- | --- | --- | --- | --- | --- |
| 1. Speech | 0.8 ± 1.0 (133) | 0.4 ± 0.8 (64) | 1.2 ± 1.1 (48) | 0.8 ± 1.0 (245) | < 0.001 |
| 2. Facial expression | 1.0 ± 0.9 (133) | 0.8 ± 0.8 (64) | 1.3 ± 0.9 (48) | 1.0 ± 0.9 (245) | 0.019 |
| 3. Rigidity | 3.9 ± 4.0 (133) | 3.2 ± 3.5 (64) | 5.2 ± 4.3 (48) | 4.0 ± 4.0 (245) | 0.025 |
| 4. Finger tapping | 2.1 ± 2.0 (132) | 2.0 ± 1.8 (63) | 2.6 ± 2.0 (48) | 2.2 ± 2.0 (243) | 0.264 |
| 5. Hand movements | 1.6 ± 1.9 (133) | 1.8 ± 1.6 (63) | 1.8 ± 2.0 (48) | 1.7 ± 1.9 (244) | 0.831 |
| 6. Pronation-supination movements of hands | 1.9 ± 2.0 (133) | 2.1 ± 1.7 (64) | 2.8 ± 2.3 (48) | 2.1 ± 2.0 (245) | 0.041 |
| 7.Toe tapping | 1.8 ± 1.9 (133) | 1.8 ± 2.0 (64) | 3.1 ± 2.2 (48) | 2.0 ± 2.1 (245) | < 0.001 |
| 8. Leg agility | 1.4 ± 1.9 (134) | 1.5 ± 1.9 (64) | 2.5 ± 2.4 (48) | 1.6 ± 2.1 (246) | 0.007 |
| 9. Arising from chair | 0.7 ± 1.1 (134) | 0.6 ± 1.0 (64) | 1.5 ± 1.5 (48) | 0.8 ± 1.2 (246) | < 0.001 |
| 10. Gait | 1.0 ± 1.1 (134) | 0.9 ± 1.1 (64) | 1.7 ± 1.3 (48) | 1.1 ± 1.2 (246) | 0.001 |
| 11. Freezing of gait | 0.6 ± 1.0 (134) | 0.7 ± 1.1 (64) | 0.9 ± 1.3 (48) | 0.7 ± 1.1 (246) | 0.332 |
| 12. Postural stability | 1.3 ± 1.3 (134) | 0.8 ± 1.2 (64) | 2.2 ± 1.4 (48) | 1.3 ± 1.4 (246) | < 0.001 |
| 13. Posture | 1.1 ± 1.0 (134) | 0.9 ± 0.9 (64) | 1.7 ± 1.2 (47) | 1.2 ± 1.1 (245) | < 0.001 |
| 14. Global spontaneity of movement (Body bradykinesia) | 1.1 ± 1.0 (134) | 1.0 ± 0.9 (64) | 1.9 ± 1.1 (47) | 1.2 ± 1.0 (245) | < 0.001 |
| 15. Postural tremor of the hands | 0.7 ± 1.1 (134) | 0.7 ± 1.4 (64) | 0.3 ± 0.8 (47) | 0.7 ± 1.2 (245) | 0.078 |
| 16. Kinetic tremor of the hands | 1.1 ± 1.5 (134) | 0.8 ± 1.3 (64) | 0.2 ± 0.6 (47) | 0.8 ± 1.4 (245) | 0.001 |
| 17. Rest tremor amplitude | 0.7 ± 1.3 (134) | 0.9 ± 1.9 (64) | 0.6 ± 1.3 (48) | 0.7 ± 1.5 (246) | 0.538 |
| 18. Constancy of rest tremor | 0.4 ± 0.6 (134) | 0.3 ± 0.6 (63) | 0.4 ± 0.9 (48) | 0.4 ± 0.6 (245) | 0.723 |

Data are mean ± standard deviation (*n*).

*p*-value: one-way analysis of variance for three-group comparisons (P-, G-, and N- group).

Abbreviations: G-group, geriatric internal medicine group; MDS-UPDRS, Japanese version of the Movement Disorder Society-Unified Parkinson’s Disease Rating Scale; N-group, neurology group; P-group, psychiatry group.

**Supplementary Table 4.** Scores for each item of the MDS-UPDRS Part II

|  | **P-group**  **(*n* = 134)** | **G-group**  **(*n* = 65)** | **N-group**  **(*n* = 49)** | **Total**  **(*N* = 248)** | ***p*-value** |
| --- | --- | --- | --- | --- | --- |
| 1. Speech | 0.8 ± 1.1 (133) | 0.5 ± 0.9 (64) | 1.2 ± 1.1 (48) | 0.8 ± 1.1 (245) | 0.004 |
| 2. Saliva and drooling | 0.6 ± 1.1 (134) | 0.3 ± 0.8 (65) | 1.0 ± 1.2 (48) | 0.6 ± 1.1 (247) | 0.004 |
| 3. Chewing and swallowing | 0.6 ± 1.0 (134) | 0.3 ± 0.7 (65) | 1.1 ± 1.3 (48) | 0.6 ± 1.0 (247) | < 0.001 |
| 4. Eating tasks | 0.7 ± 0.9 (132) | 0.4 ± 0.7 (64) | 1.1 ± 1.0 (48) | 0.7 ± 0.9 (244) | < 0.001 |
| 5. Dressing | 1.0 ± 1.0 (132) | 0.8 ± 1.0 (64) | 1.6 ± 1.2 (48) | 1.1 ± 1.1 (244) | < 0.001 |
| 6. Hygiene | 1.0 ± 1.1 (134) | 0.7 ± 0.9 (64) | 1.3 ± 1.2 (48) | 1.0 ± 1.1 (246) | 0.019 |
| 7. Handwriting | 1.1 ± 1.1 (134) | 0.8 ± 1.1 (64) | 1.6 ± 1.3 (48) | 1.1 ± 1.2 (246) | 0.001 |
| 8. Doing hobbies and other activities | 1.3 ± 1.3 (133) | 1.0 ± 1.3 (64) | 2.4 ± 1.6 (48) | 1.4 ± 1.4 (245) | < 0.001 |
| 9. Turning in bed | 0.5±0.9 (133) | 0.2 ± 0.4 (64) | 1.3 ± 1.3 (48) | 0.5 ± 1.0 (245) | < 0.001 |
| 10. Tremor | 0.7 ± 0.8 (133) | 0.2 ± 0.6 (65) | 0.6 ± 0.6 (48) | 0.5 ± 0.8 (246) | < 0.001 |
| 11. Getting out of bed, a car, or a deep chair | 1.0 ± 1.1 (133) | 0.7 ± 1.0 (65) | 1.9 ± 1.4 (47) | 1.1 ± 1.2 (245) | < 0.001 |
| 12. Walking and balance | 1.2 ± 1.2 (133) | 1.1 ± 1.3 (65) | 2.0 ± 1.3 (48) | 1.3 ± 1.3 (246) | < 0.001 |
| 13. Freezing | 0.7 ± 1.1 (133) | 0.6 ± 1.1 (65) | 1.1 ± 1.5 (48) | 0.8 ± 1.2 (246) | 0.046 |

Data are mean ± standard deviation (*n*).

*p*-value: one-way analysis of variance for three-group comparisons (P-, G-, and N- group).

Abbreviations: G-group, geriatric internal medicine group; MDS-UPDRS, Japanese version of the Movement Disorder Society-Unified Parkinson’s Disease Rating Scale; N-group, neurology group; P-group, psychiatry group.

**Supplementary Table 5.** Symptom domains and individual symptoms for each domain that caused patients the most distress

|  | **Symptoms that caused the patients the most distress** | | |
| --- | --- | --- | --- |
|  | **P-group**  **(*n* = 134)** | **G-group**  **(*n* = 65)** | **N-group**  **(*n* = 49)** |
| Total number of cognitive impairments | 22 (16.4) | 11 (16.9) | 5 (10.2) |
| Memory impairment  Disorientation  Executive dysfunction  Attention dysfunction  Fluctuation cognition  Visuospatial dysfunction  Other cognitive impairment | 16 (11.9)  1 (0.7)  2 (1.5)  2 (1.5)  1 (0.7)  0 (0.0)  0 (0.0) | 5 (7.7)  1 (1.5)  1 (1.5)  2 (3.1)  1 (1.5)  1 (1.5)  0 (0.0) | 1 (2.0)  0 (0.0)  0 (0.0)  0 (0.0)  0 (0.0)  0 (0.0)  4 (8.2) |
| Total number of parkinsonism | 19 (14.2) | 8 (12.3) | 19 (38.8) |
| Bradykinesia/akinesia  Rigidity  Action tremor  Rest tremor  Postural instability  Gait disturbance  Freezing of gait  Abnormal posture  Salivation  Fall  Dysphagia | 8 (6.0)  0 (0.0)  1 (0.7)  1 (0.7)  2 (1.5)  3 (2.2)  1 (0.7)  2 (1.5)  0 (0.0)  1 (0.7)  0 (0.0) | 1 (1.5)  0 (0.0)  0 (0.0)  0 (0.0)  2 (3.1)  0 (0.0)  1 (1.5)  2 (3.1)  2 (3.1)  0 (0.0)  0 (0.0) | 8 (16.3)  1 (2.0)  2 (4.1)  0 (0.0)  0 (0.0)  3 (6.1)  1 (2.0)  2 (4.1)  1 (2.0)  1 (2.0)  0 (0.0) |
| Total number of psychiatric symptoms | 11 (8.2) | 5 (7.7) | 2 (4.1) |
| Delusions  Visual hallucinations  Hallucinations otter than visual hallucinations  Agitation/aggression  Depression  Anxiety  Apathy  Disinhibition  Aberrant motor behavior  Negativism  Delirium  Other psychiatric symptoms | 1 (0.7)  6 (4.5)  1 (0.7)  0 (0.0)  1 (0.7)  2 (1.5)  0 (0.0)  0 (0.0)  0 (0.0)  0 (0.0)  0 (0.0)  0 (0.0) | 0 (0.0)  0 (0.0)  1 (1.5)  0 (0.0)  1 (1.5)  2 (3.1)  0 (0.0)  0 (0.0)  0 (0.0)  0 (0.0)  0 (0.0)  1 (1.5) | 0 (0.0)  1 (2.0)  1 (2.0)  0 (0.0)  0 (0.0)  0 (0.0)  0 (0.0)  0 (0.0)  0 (0.0)  0 (0.0)  0 (0.0)  0 (0.0) |
| Total number of eating behavior-related problems | 2 (1.5) | 1 (1.5) | 2 (4.1) |
| Loss of appetite  Increase in appetite  Weight loss  Weight gain  Food refusal  Eating non-edible things  Unbalanced diet | 1 (0.7)  0 (0.0)  0 (0.0)  1 (0.7)  0 (0.0)  0 (0.0)  0 (0.0) | 0 (0.0)  0 (0.0)  1 (1.5)  0 (0.0)  0 (0.0)  0 (0.0)  0 (0.0) | 0 (0.0)  0 (0.0)  1 (2.0)  1 (2.0)  0 (0.0)  0 (0.0)  0 (0.0) |
| Total number of sleep-related disorder | 8 (6.0) | 2 (3.1) | 3 (6.1) |
| Rapid eye movement sleep behavior disorder  Daytime somnolence  Day-night reversal  Night-time sleep disorder  Sudden sleep  Restless legs syndrome  Periodic limb movement disorder | 2 (1.5)  1 (0.7)  0 (0.0)  5 (3.7)  0 (0.0)  0 (0.0)  0 (0.0) | 2 (3.1)  0 (0.0)  0 (0.0)  0 (0.0)  0 (0.0)  0 (0.0)  0 (0.0) | 1 (2.0)  0 (0.0)  1 (2.0)  1 (2.0)  0 (0.0)  0 (0.0)  0 (0.0) |
| Total number of autonomic dysfunctions | 15 (11.2) | 12 (18.5) | 6 (12.2) |
| Orthostatic hypotension  Disturbance of sweating  Constipation  Night-time dysuria  Daytime dysuria  Syncope  Dizziness | 0 (0.0)  1 (0.7)  9 (6.7)  2 (1.5)  3 (2.2)  0 (0.0)  0 (0.0) | 0 (0.0)  1 (1.5)  7 (10.8)  3 (4.6)  1 (1.5)  0 (0.0)  0 (0.0) | 1 (2.0)  0 (0.0)  3 (6.1)  1 (2.0)  1 (2.0)  0 (0.0)  0 (0.0) |
| Total number of sensory disorders | 0 (0.0) | 0 (0.0) | 0 (0.0) |
| Dysosmia | 0 (0.0) | 0 (0.0) | 0 (0.0) |
| Total number of invalid answers | 57 (42.5) | 26 (40.0) | 12 (24.5) |
| Do not know  Unanswered  Multiple answers | 17 (12.7)  7 (5.2)  33 (24.6) | 7 (10.8)  4 (6.2)  15 (23.1) | 5 (10.2)  0 (0.0)  7 (14.3) |

Data are *n* (%).

Abbreviations: G-group, geriatric internal medicine group; N-group, neurology group; P-group, psychiatry group.

**Supplementary Table 6.** Symptom domains and individual symptoms for each domain that caused caregivers the most distress

|  | **Symptoms that caused the caregivers the most distress** | | |
| --- | --- | --- | --- |
|  | **P-group**  **(*n* = 134)** | **G-group**  **(*n* = 65)** | **N-group**  **(*n* = 49)** |
| Total number of cognitive impairments | 23 (17.2) | 25 (38.5) | 3 (6.1) |
| Memory impairment  Disorientation  Executive dysfunction  Attention dysfunction  Fluctuation cognition  Visuospatial dysfunction  Other cognitive impairment | 7 (5.2)  0 (0.0)  5 (3.7)  2 (1.5)  5 (3.7)  0 (0.0)  4 (3.0) | 14 (21.5)  1 (1.5)  6 (9.2)  0 (0.0)  2 (3.1)  1 (1.5)  1 (1.5) | 2 (4.1)  0 (0.0)  0 (0.0)  0 (0.0)  0 (0.0)  0 (0.0)  1 (2.0) |
| Total number of parkinsonism | 14 (10.4) | 0 (0.0) | 17 (34.7) |
| Bradykinesia/akinesia  Rigidity  Action tremor  Rest tremor  Postural instability  Gait disturbance  Freezing of gait  Abnormal posture  Salivation  Fall  Dysphagia | 6 (4.5)  3 (2.2)  1 (0.7)  0 (0.0)  1 (0.7)  2 (1.5)  0 (0.0)  0 (0.0)  0 (0.0)  1 (0.7)  0 (0.0) | 0 (0.0)  0 (0.0)  0 (0.0)  0 (0.0)  0 (0.0)  0 (0.0)  0 (0.0)  0 (0.0)  0 (0.0)  0 (0.0)  0 (0.0) | 5 (10.2)  0 (0.0)  1 (2.0)  1 (2.0)  3 (6.1)  2 (4.1)  1 (2.0)  1 (2.0)  2 (4.1)  1 (2.0)  0 (0.0) |
| Total number of psychiatric symptoms | 34 (25.4) | 12 (18.5) | 6 (12.2) |
| Delusions  Visual hallucinations  Hallucinations otter than visual hallucinations  Agitation/aggression  Depression  Anxiety  Apathy  Disinhibition  Aberrant motor behavior  Negativism  Delirium  Other psychiatric symptoms | 8 (6.0)  12 (9.0)  3 (2.2)  7 (5.2)  1 (0.7)  1 (0.7)  0 (0.0)  0 (0.0)  0 (0.0)  0 (0.0)  0 (0.0)  2 (1.5) | 0 (0.0)  3 (4.6)  1 (1.5)  5 (7.7)  0 (0.0)  0 (0.0)  0 (0.0)  0 (0.0)  2 (3.1)  0 (0.0)  0 (0.0)  1 (1.5) | 1 (2.0)  2 (4.1)  0 (0.0)  2 (4.1)  0 (0.0)  0 (0.0)  0 (0.0)  0 (0.0)  1 (2.0)  0 (0.0)  0 (0.0)  0 (0.0) |
| Total number of eating behavior-related problems | 3 (2.2) | 1 (1.5) | 1 (2.0) |
| Loss of appetite  Increase in appetite  Weight loss  Weight gain  Food refusal  Eating non-edible things  Unbalanced diet | 2 (1.5)  0 (0.0)  1 (0.7)  0 (0.0)  0 (0.0)  0 (0.0)  0 (0.0) | 0 (0.0)  1 (1.5)  0 (0.0)  0 (0.0)  0 (0.0)  0 (0.0)  0 (0.0) | 0 (0.0)  0 (0.0)  0 (0.0)  1 (2.0)  0 (0.0)  0 (0.0)  0 (0.0) |
| Total number of sleep-related disorder | 6 (4.5) | 4 (6.2) | 3 (6.1) |
| Rapid eye movement sleep behavior disorder  Daytime somnolence  Day-night reversal  Night-time sleep disorder  Sudden sleep  Restless legs syndrome  Periodic limb movement disorder | 3 (2.2)  0 (0.0)  0 (0.0)  3 (2.2)  0 (0.0)  0 (0.0)  0 (0.0) | 3 (4.6)  0 (0.0)  0 (0.0)  0 (0.0)  0 (0.0)  1 (1.5)  0 (0.0) | 0 (0.0)  2 (4.1)  0 (0.0)  0 (0.0)  1 (2.0)  0 (0.0)  0 (0.0) |
| Total number of autonomic dysfunctions | 4 (3.0) | 6 (9.2) | 8 (16.3) |
| Orthostatic hypotension  Disturbance of sweating  Constipation  Night-time dysuria  Daytime dysuria  Syncope  Dizziness | 0 (0.0)  0 (0.0)  2 (1.5)  2 (1.5)  0 (0.0)  0 (0.0)  0 (0.0) | 0 (0.0)  0 (0.0)  3 (4.6)  2 (3.1)  0 (0.0)  1 (1.5)  0 (0.0) | 1 (2.0)  0 (0.0)  3 (6.1)  3 (6.1)  0 (0.0)  0 (0.0)  1 (2.0) |
| Total number of sensory disorders | 0 (0.0) | 0 (0.0) | 0 (0.0) |
| Dysosmia | 0 (0.0) | 0 (0.0) | 0 (0.0) |
| Total number of invalid answers | 50 (37.3) | 17 (26.2) | 11 (22.4) |
| Do not know  Unanswered  Multiple answers | 9 (6.7)  3 (2.2)  38 (28.4) | 0 (0.0)  0 (0.0)  17 (26.2) | 1 (2.0)  2 (4.1)  8 (16.3) |

Data are *n* (%).

Abbreviations: G-group, geriatric internal medicine group; N-group, neurology group; P-group, psychiatry group.
